# HIV associated lymphoma: latest updates from 2023 ASH annual meeting

**DOI:** 10.1186/s40164-024-00530-6

**Published:** 2024-07-05

**Authors:** Chaoyu Wang, Qing Xiao, Xiaomei Zhang, Yao Liu

**Affiliations:** Department of Hematology, Chongqing Key Laboratory of Translational Research for Cancer Metastasis and Individualized Treatment, Chongqing University Cancer Hospital, Chongqing, 400030 China

## Abstract

The incidence, clinical characteristics, and prognostic factors of HIV-associated lymphoma remain poorly defined compared to HIV-negative lymphoma. Currently, there are no standard guidelines for treatment of these patients. We summarized several latest reports of HIV associated lymphoma from the 2023 ASH Annual Meeting (ASH2023).

## To the editor

People living with human immunodeficiency virus (HIV) infection (PLWH) are at increased risk for lymphoma. Since the widespread use of combined antiretroviral therapy (cART), the incidence of most types of non-Hodgkin lymphoma affecting PLWH have decreased, but the incidence of HL has increased [1, 2]. We summarized several latest reports of HIV associated lymphoma from the 2023 ASH Annual Meeting (ASH2023).

### Outcomes of HIV associated DLBCL during the antiretroviral therapy era

A retrospective population-based cohort [3] was conducted using US population-based cancer registry data among patients aged 15–79 years with newly diagnosed DLBCL from 2010 to 2017. A total of 26,905 DLBCL patients were identified, which including 1,030 patients with HIV. Compared to patients without HIV, HIV associated DLBCL were younger (median age at diagnosis 48 years vs. 62 years), predominantly male (85% vs. 56%), and more likely to have advanced-stage disease at diagnosis (71% vs. 56%). With a median follow-up of 67 months, 5-year OS was particularly low among HIV associated lymphoma than HIV negative lymphoma patients. This study highlights worse outcomes among HIV associated DLBCL compared to patients without HIV in the modern era of cART.

### Epidemiology and outcomes of HIV associated lymphoma during the antiretroviral therapy era

Another retrospective study [4], Kathryn Kline enrolling 181 HIV-associated lymphoma patients, which including 160 patients during 2000–2013, and 51 patients during 2014–2021. Diffuse large B-cell lymphoma (DLBCL) was the most common type of lymphoma throughout the study period, representing 54 of 131 (41%) cases during 2000–2013 and 29 of 50 (58%) of cases during 2014–2021. Hodgkin lymphoma (HL) was the 2 -most common lymphoma, comprising 18% of cases during 2000–2013 and 16% during 2014–2021. The proportion of patients receiving some lymphoma treatment was 85% during 2000–2013 and 96% during 2014–2021, including 92% who received chemotherapy and/or monoclonal antibodies. Two year overall survival was 41% during 2000–2013 and 50% during 2014–2021 (*p* = 0.44) (Table 1).

**Table 1:** Epidemiology trends of HIV-associated lymphoma

Histotype	2000–2013 N = 131	2000–2013 On cART, N = 28	2000–2013 No cART, n = 103	2014–2021 N = 50	2014–2021 On cART, N = 30	2014–2021 No cART, N = 20	References
HL	18%	36%	14%	16%	17%	15%	[4]
DLBCL	41%	32%	44%	58%	63%	50%	
BL	21%	18%	8%	6%	0	15%	
PCNSL	5%	4%	22%	4%	3%	5%	
PBL	6%	0	8%	2%	3%	0	
PEL	2%	4%	1%	0	0	0	
Other	6%	8%	6%	14%	10%	20%	

## Prognostic factors of HIV associated HL treated with ABVD

Retrospective multicentric study [5] of patients with HIV-associated HL in 9 hospitals from Spain during 1995 to 2022. All patients were treated with ABVD and cART. Ninety patients were retrospectively analyzed with a median follow up of 5.89 years. In the univariate analysis, bone marrow involvement and monocytes count ≥ 0.6 × 10^9/L were associated with shorter overall survival (OS) and progression free survival (PFS) probabilities. A lymphocyte/monocyte (L/M) ratio < 1.09 was associated with shorter PFS probabilities. By multivariable analysis, only monocyte count ≥ 0.6 × 10^9/L emerged as an unfavorable prognostic factor for OS and PFS. High monocyte count is a strong prognostic factor which can be used in HIV-associated HL. Most HIV-associated HL patients present an advanced stage at diagnosis or a localized stage with unfavorable prognosis factors.

### Outcomes of HIV associated HL treated with PD-1 blockade

Another retrospective study [6], Kathryn Lurain enrolling 23 HIV-associated HL patients from 12 institutions in the United States. 19 (83%) had advanced stage disease, and in 20 pts where EBV status of the tumor was known, all were positive. Nine (39%) pts had primary refractory disease. Fourteen (61%) pts received doxorubicin, bleomycin, vinblastine, and dacarbazine (ABVD) as 1 -line therapy. Seventeen (74%) pts received PD-1 monotherapy, 5 (22%) received nivolumab plus brentuximab vedotin, and 1 received nivolumab plus ifosfamide, carboplatin, and etoposide. All pts received antiretroviral therapy during treatment. Median baseline CD4 was 155 cells/µL (IQR: 66–297). Median CD4 increased to 310 at the end of PD-1 treatment (*P* = 0.0084). The median number of PD-1 cycles was 6 (IQR: 4–21). Three (13%) pts had irAEs (all grade 3): hypothyroidism, autoimmune pancreatitis, and pneumonitis. None required PD-1 discontinuation. The ORR was 83% in the overall cohort. Sixteen pts (70%) had CR, 3 (13%) PR, and 4 (17%) PD as best response. At data cut-off, 17 pts (74%) were alive and 6 (26%) had died due to HIV-HL. Median OS was 23.6 months in the overall cohort. In HIV-HL, PD-1 blockade was safe, had a high ORR and CR rate, and allowed for immune reconstitution in pts across a wide range of CD4 counts and HIV viral loads. These data add to the evidence that anti-PD-1 agents are effective in PWH and should be used in HIV-HL.

### 11q aberration is not a rare event in HIV-associated lymphoma

Of the 144 cases [7], 123 (85%) were evaluable by FISH, including 76 DLBCL, 10 BL, 14 primary effusion lymphoma (PEL), 7 plasmablastic lymphomas, 1 marginal zone lymphomas and 15 polymorphic lymphoproliferative lesions. Four abnormal patterns were seen in 39 cases (32%): polysomy (≥ 3 copies of chromosome 11), 11q23q24 amplification (≥ 10 copies), variant or atypical 11q aberration (11q24 loss only) and classic or typical 11q aberration (11q23 gain and 11q24 loss). This results demonstrate that chromosome 11 abnormalities are not an uncommon finding in HIV positive lymphoproliferative lesions (Table 2).

**Table 2:** Updates on survival for HIV-associated lymphoma

Author	Study Type	Regimen	Patients	ORR	CR	Survival	References
Bryan Valcarcel	Retrospective	R-CHOP	DLBCL, N = 1030	--	--	5-year OS: 45%-53%	[3]
Kathryn Kline	Retrospective	Chemotherapy and/or monoclonal anti bodies	HIV-assosiated lymphoma, N = 181	--	--	2-year OS:	[4]
			(DLBCL, N = 83; HL, N = 32; PCNSL, N = 9;			41%(2000–2013);	
			BL, N = 31; PEL, N = 2; PBL, N = 9;Other, N = 15			50%(2014–2021)	
Maria Huguet	Retrospective	ABVD	HL, N = 19	--	--	5-year OS: 93%	[5]
						(monocyte count < 0.6 x10^^^9/LV)	
Kathryn Lurain	Retrospective	PD-1	HL, N = 23	83%	70%	Median OS: 23.6 months	[6]

Abbreviations: DLBCL: diffuse large B cell lymphoma; HL: Hodgkin lymphoma; PCNSL: primary central nervous system lymphoma; BL: Burkitt’s lymphoma;PEL: primary effusion lymphoma; PBL: Plasmoblastic lymphoma; CR: complete response; ORR: overall response rate; PFS: progression free survival; OS: overall survival; cART: combined antiretroviral therapy

HIV-associated lymphoma has significantly improved due to the wide use of cART. Using similar curative chemoimmunotherapy regimens as the ones given in HIV negative lymphoma patients, has also improved these outcomes. More prospective randomized clinical trials with HIV-associated lymphoma are required in the future. **HIV associated lymphoma should also be considered appropriate potential participants for future clinic trials, including bispecifc antibodies and CAR-T cell therapy.**

## Data Availability

No datasets were generated or analysed during the current study.

## Abbreviations

ASH: American Society of Hematology
HIV: Human immunodeficiency virus
DLBCL: Diffuse large B cell lymphoma
HL: Hodgkin lymphoma
PEL: Primary effusion lymphoma
CR: Complete response
ORR: Overall response rate
PFS: Progression free survival
OS: Overall survival
cART: Combined antiretroviral therapy
